# Direct and indirect effects of roads on space use by jaguars in Brazil

**DOI:** 10.1038/s41598-021-01936-6

**Published:** 2021-11-19

**Authors:** Rafaela Cobucci Cerqueira, Oscar Rodríguez de Rivera, Jochen A. G. Jaeger, Clara Grilo

**Affiliations:** 1 Departamento de Biologia, Universidade Federal de Lavras, Câmpus Universitário, Caixa Postal 3037, Lavras, Minas Gerais CEP 37200-000 Brazil; 2 School of Mathematics, Statistics and Actuarial Science, University of Kent, Sibson, Park Wood Rd, Canterbury, CT2 7FS UK; 3 Department of Geography, Planning and Environment, Concordia University Montreal, 1455 de Maisonneuve Blvd. W., Suite H1255, Montréal, QC H3G 1M8 Canada; 4 CESAM - Centro de Estudos do Ambiente e do Mar, Departamento de Biologia Animal, Faculdade de Ciências, Universidade de Lisboa, 1749-016 Lisboa, Portugal

**Keywords:** Conservation biology, Ecology, Ecological modelling

## Abstract

Roads pose an imminent threat to wildlife directly through mortality and changes in individual behavior, and also indirectly through modification of the amount and configuration of wildlife habitat. However, few studies have addressed how these mechanisms interact to determine species response to roads. We used structural equation modeling to assess direct and indirect effects (via landscape modification) of roads on space use by jaguars in Brazil, using radio-tracking data available from the literature. We fit path models that directly link jaguars’ space use to roads and to land cover, and indirectly link jaguars’ space use to roads through the same land cover categories. Our findings show that space use by jaguars was not directly affected by roads, but indirect effects occurred through reductions in natural areas on which jaguars depend, and through urban sprawl. Males´ space use, however, was not negatively influenced by urban areas. Since jaguars seem to ignore roads, mitigation should be directed to road fencing and promoting safe crossings. We argue that planners and managers need to much more seriously take into account the deforestation and the unbridled urban expansion from roads to ensure jaguar conservation in Brazil.

## Introduction

Guided primarily by the argument of socio-economic development, investments in road expansion worldwide have never before been so high as today^1,2^. In Brazil, the government is planning to add nearly 129,000 km to the existing 1.7 million kilometers of roads in the next 20 years^3,4^. Many of the planned roads will be built in areas of high biodiversity value such as the biomes Amazon, Cerrado, and Atlantic Forest^5,6^.

Roads are among the most important impacts on wildlife populations and species distribution^7,8^. Their effects can be direct as they cause mortality through collision with vehicles, e.g., by attraction to suitable roadside vegetation for refuge or predation^9,10^, and changes in spatial behavior, e.g., by avoidance of traffic noise and light^11,12^. Road effects on wildlife can also be indirect by promoting changes in the landscape as they remove natural vegetation and bisect large contiguous areas^13,14^. Roads are known to facilitate the urban sprawl, deforestation, intensive farming, and illegal human activities such as poaching^5,9^. Habitat loss due to landscape changes caused by human activities negatively affects many species’ occurrence and abundance and species richness^15–17^.

Road ecology research has long focused on the impacts of infrastructure on wildlife behavior, occurrence, abundance, and persistence^8,18^. Such studies are typically conducted to evaluate how roads and traffic affect wildlife (e.g.,^11,19^) or to analyze how roads change landscape composition and the spatial configuration of wildlife habitat (e.g.,^20^) without considering how these two mechanisms interact when wildlife populations respond to roads.

Apex predators such as the jaguar (*Panthera onca*) are particularly vulnerable to the negative effects of roads due to low population densities, large spatial requirements, and low reproductive rates^21,22^. The jaguar is the largest felid in the Americas^23^ and has been extirpated from more than 50% of its historical range (from southwestern United States to Central Argentina^24^). As a result, it is now ranked 15th among large mammal species with the greatest range contractions due to anthropogenic effects globally^25^. Several studies have assessed the behavior of jaguars in response to roads and land cover^26–29^. They showed that jaguars move preferentially in undisturbed natural areas far from roads and other human occupations such as agricultural lands and areas of high human population density^30–32^. However, no study has analyzed if the effects of roads are direct or indirect through the modification of jaguars’ habitat.

**Figure 1:**
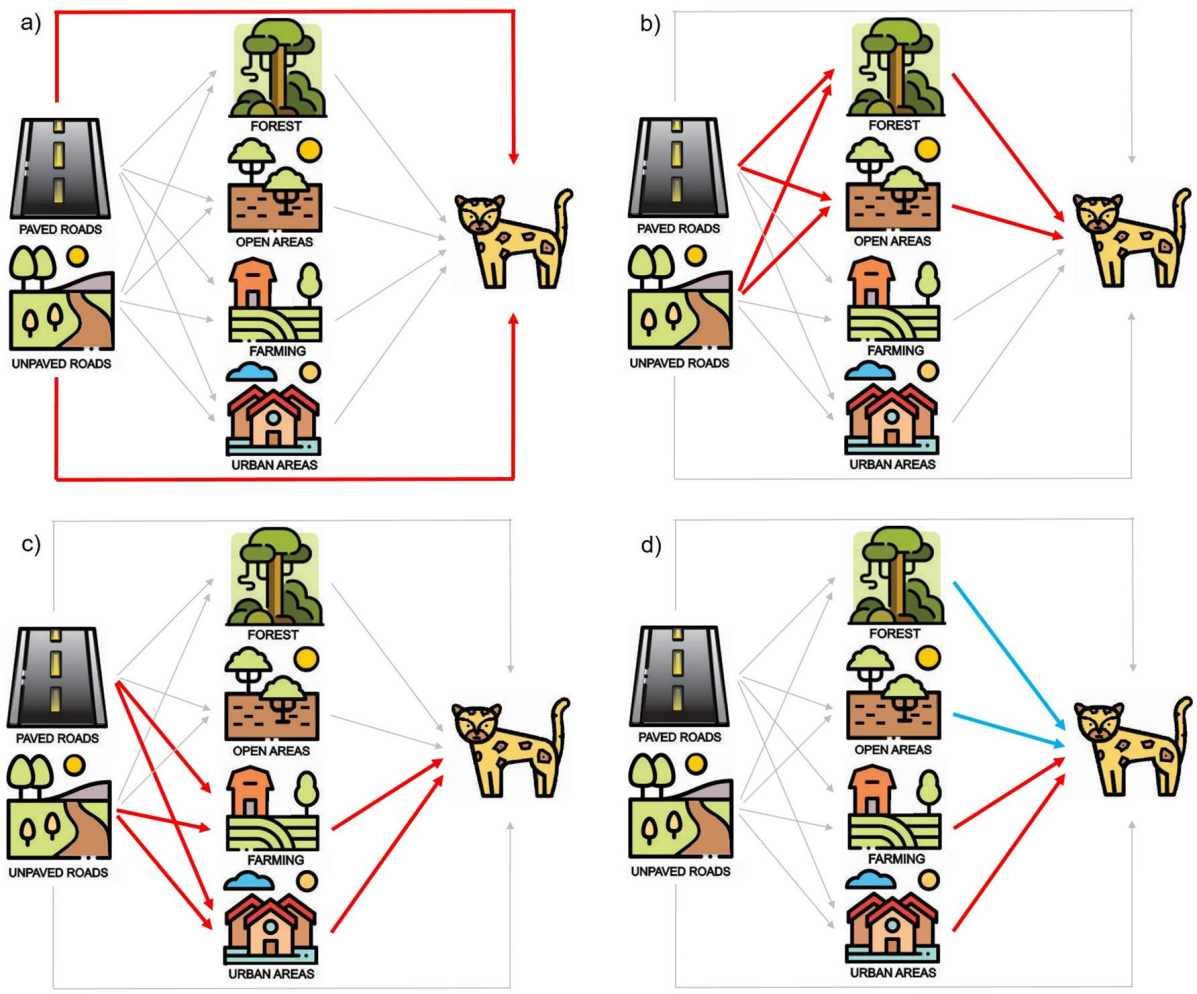
Conceptual framework to assess the direct and indirect effects of roads on jaguars’ space use according to four hypotheses: (**a**) Space use by jaguars is predominantly affected directly by roads; (**b**) space use by jaguars is strongly affected indirectly by roads via the effects of roads on natural areas (i.e., roads promote a reduction in forest and open areas and consequently have a negative effect on jaguars’ use of habitat); (**c**) space use by jaguars is primarily affected indirectly by roads via the effects of roads on human-dominated areas (i.e., roads promote an increase of farming and urban areas and consequently have a negative effect on jaguars’ use of habitat); (**d**) space use by jaguars is mostly affected directly by land cover independently of roads (i.e., forest and open areas influence the jaguars’ space use while farming and urban areas affect them negatively). Colored arrows denote expected positive (blue) or negative (red) effects of variables on jaguars’ space use. Direct effects of variables on jaguars’ space use are depicted by solid arrows, while indirect effects are depicted by dashed arrows. To avoid duplicate figures, the conceptual model is presented with paved and unpaved roads together, but separate models were generated for each.

**Figure 2:**
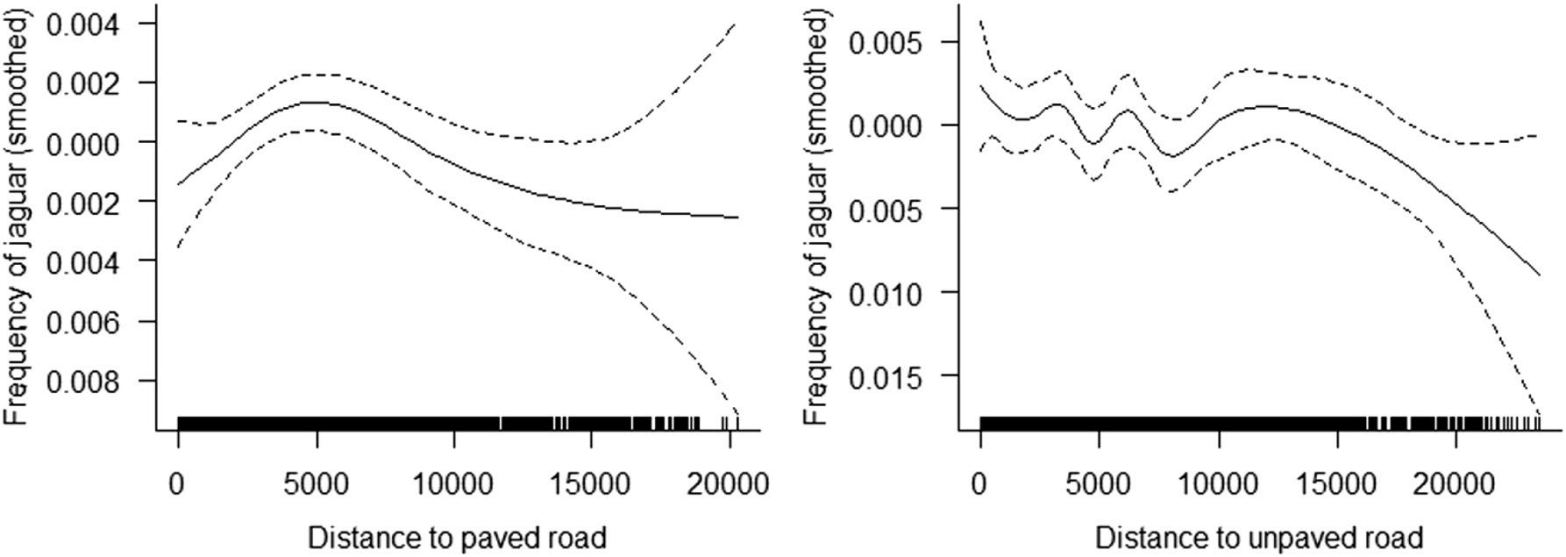
Smoothed curves showing the relationships between jaguars’ space use (measured as frequency of jaguar locations/number of locations per day) and distance (m) to paved and unpaved roads. The smoother is centred around zero. Dashed lines represent 95% confidence intervals.

**Figure 3:**
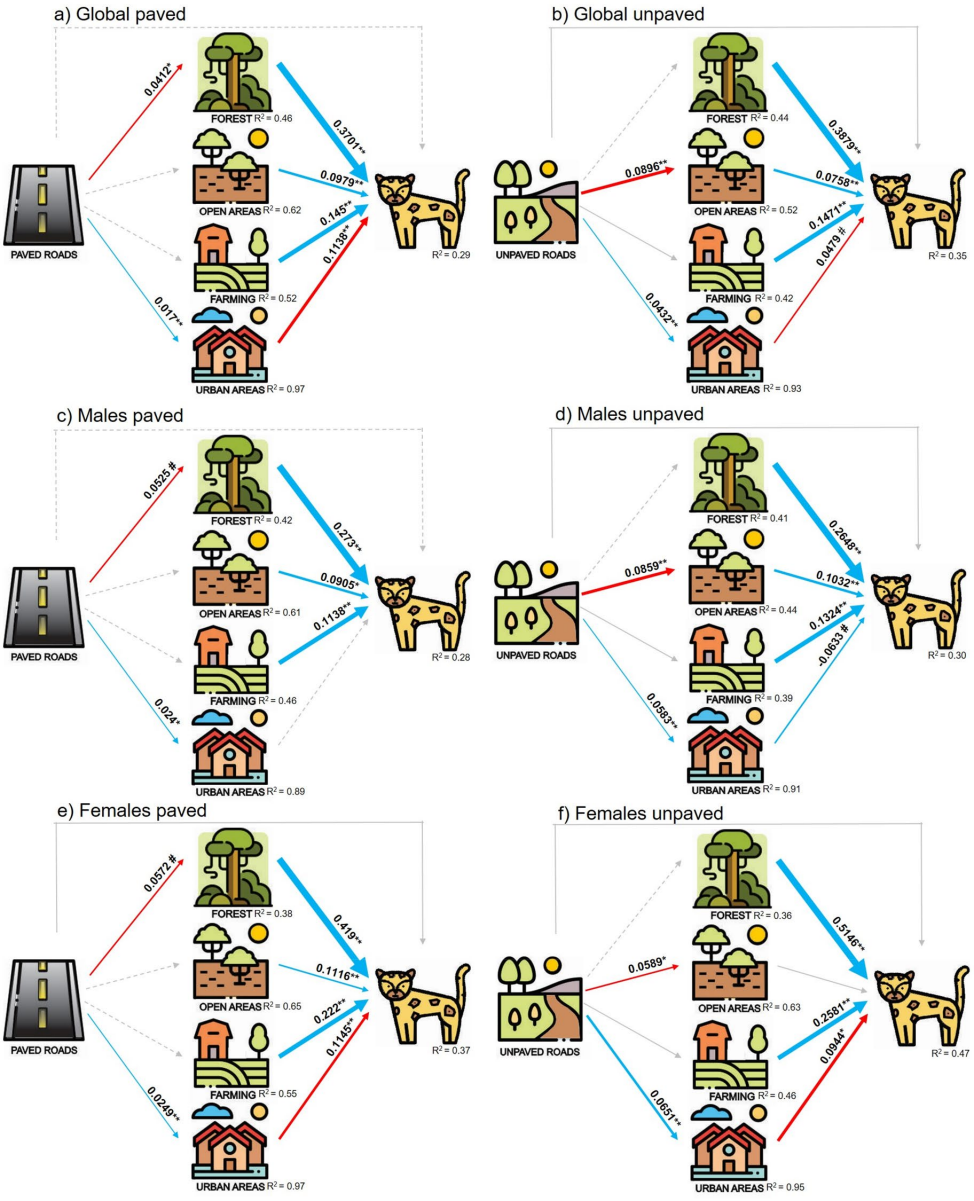
Path diagrams representing the effects of roads and land cover on jaguars’ space use for the global model (**a**, **b**), for males (**c**, **d**), and females (**e**, **f**) for paved and unpaved roads, respectively. Arrows represent unidirectional relationships among variables. Colored arrows indicate positive (blue) and negative (red) significant effects and gray arrows denote non-significant positive (solid) or negative (dashed) paths. The numbers associated with the arrows provide the standardized coefficients and the width of the arrows refers to the size of the coefficients of significant effects. Numbers below the response variables are pseudo-*R*-squared values. Note that for those variables measured as distances (roads and urban areas), a negative effect occurred when the coefficient is positive, and vice-versa, except for the effect of roads on urban areas which are both measured as distances (Supplementary Table S2 online). # marginally significant effect with *p-*value < 0.1, * *p*-value < 0.05, and **p-value < 0.01.

The main goal of this study was to disentangle the direct and indirect effects of roads on jaguars’ space use throughout their range in Brazil. We used structural equation modelling, an approach that combines multiple predictor and response variables in a single causal network^33^. We fit path models^34^ that link directly jaguars’ space use to roads and to four land cover categories, namely, forest (natural dense vegetation and secondary forest), natural open areas (savanna formations and grasslands, hereafter, open areas), farming (pasture and/or agriculture) and urban areas, and also link indirectly jaguars’ space use to roads through the same land cover categories. We specifically tested four hypotheses: (1) jaguars prefer areas far from roads primarily because of the direct effect of roads (Fig. 1a); (2) jaguars prefer areas far from roads because roads are associated with a reduction in the amount of forest and open areas that favor their occurrence, i.e., the indirect effects of roads via natural areas are predominant (Fig. 1b); (3) jaguars prefer areas far from roads because roads promote the expansion of farming and urbanized areas that impair the occurrence of jaguars, i.e., the indirect effect of roads via human-dominated areas is predominant (Fig. 1c); (4) space use by jaguars is primarily determined by land cover rather than roads, i.e., the direct effects of land cover are predominant (Fig. 1d). This study intends to contribute to a more comprehensive and integrated understanding of species’ responses towards roads to promote effective measures for jaguar conservation in roaded landscapes.

## Methods

### Jaguar data and study area

We used a large dataset of jaguar locations tracked by GPS technology in Brazil from Morato et al.^35^ to analyse the relationships between jaguars’ space use and roads and land cover. The data were from 82 individuals monitored by eleven studies encompassing different terrestrial biomes in Brazil (Supplementary Fig. S1 online). The jaguar locations were distributed in 15 areas (Supplementary Fig. S1 online). We delimited each area using kernel density estimation at the 95% isopleth using the locations of all individuals. Because we were interested in the influence of roads on jaguars and on landscape structure, we then selected only the areas that were intersected by either paved or unpaved roads (Supplementary Fig. S1 online).

To estimate space use by jaguars, we translated jaguar locations into frequency values. For each individual, we calculated the relative frequency of locations (number of locations of an individual divided by the total number of sampling days of that individual) in a grid with cell size of 1 km × 1 km. For cells with more than one individual, we estimated the average frequency. To control for differences among studies regarding the sampling frequency, we used only one random location per individual from every 24-h period (Supplementary Table S1 online). Lastly, we selected “zero cells” (cells not used by jaguars) to represent one third of the number of cells with information on frequency of jaguar locations. Grid cells exhibiting the highest frequencies were the most visited by jaguars.

### Environmental data

We obtained the road network (paved and unpaved roads) from OpenStreetMap (Geofabrik^36^—http://www.geofabrik.de) and land cover variables from MapBiomas (collection 2, Projeto MapBiomas^37^ http://mapbiomas.org). We relied on the map of 2015 of MapBiomas because most of the jaguar data were from between 2008 and 2015 (Supplementary Table S1 online). We aggregated and reclassified land cover into four categories that were reported to influence jaguar occurrence^38^: forest (natural dense vegetation and secondary forest), open areas (savanna formations and grasslands), farming (pasture and/or agriculture) and urban areas. For each 1 km × 1 km cell, we estimated the variables as follows: distance between the centroid of the cell and the nearest road (paved and unpaved separately, located within or outside the cell); distance between the centroid of the cell and the nearest urban area (located within or outside the cell); proportion of forest, open areas, and farming within the cell. All variables were calculated using ArcGIS 10.3^39^.

### Data analysis

We inspected for a threshold distance above which paved and unpaved roads may not have any influence on jaguars and analysed direct and indirect effects of roads only for cells within the distance threshold determined. To find this threshold we explored generalized additive models (GAMs) using the package mgcv in R^40^.

We estimated direct and indirect (via land cover) effects of roads on jaguars’ space use using piecewise Structural Equation Modelling (SEM^41^). SEM is a probabilistic approach commonly used to study ecological systems that are driven by interconnected processes^42^ as it that allows for using multiple predictor and response variables to assess simultaneous influences and responses in a single network^33^. It differs from other modelling approaches as it attempts to model causal relations between multiple variables known to be involved in a complex system, thus allowing correlations to reflect causal relationships^33,43^. SEM is usually represented with path diagrams that evaluate the direct and indirect effects of hypothesized causal relationships^44^. In piecewise SEM, a path diagram is translated to a set of linear (structural) and individual equations^34,41^. We fitted path models that link directly jaguars’ space use to roads and to four land-cover categories (Fig. 1).

We assessed whether paved and unpaved roads affect jaguars’ space use directly or indirectly through land cover. In the piecewise SEM, the space use by jaguars (both males and females, or males, or females) was the main variable to be explained and the five other variables (four land-cover variables and type of road (unpaved or paved)) were linked in causal relationships^34^, Fig. 1; these and other hypothesized links are presented in Supplementary Table S2 online, as well as the possible mechanisms explaining the links). Specifically, we used simultaneous autoregressive (SAR) models^45,46^ to account for spatial autocorrelation of jaguar data and calculated Generalized R-square values (see details in Supplementary Text S1 online). We applied SEM in six models (see Fig. 3): (a) both genders ~ 4 land covers + paved roads (we called it Global paved model); (b) both genders ~ 4 land covers + unpaved roads (Global unpaved), (c) males ~ 4 land covers + paved roads (Males paved); (d) males ~ 4 land covers + unpaved roads (Males unpaved), (e) females ~ 4 land covers + paved roads (Females paved), and (f) females 4 land covers + unpaved roads (Females unpaved).

We did not perform any model selection process because we wanted to assess the relationships between roads, land cover variables (natural and human-dominated), and jaguars’ space use. All variables were scaled (*x*-mean(*x*))/sd(*x*)) prior to the analysis to make coefficients comparable. An initial Spearman’s rank correlation was performed on the dataset to check for multicollinearity, and since none of the variables were highly correlated (all r < 0.56), all of them were included in the models (Supplementary Fig. S2 online).

Output model coefficients (path coefficients) allow for a comparison of the relative importance of direct and indirect causal links. The indirect effect of roads on jaguars’ space use was obtained by multiplying the patch coefficient linking roads to the land cover variables and the path coefficient linking the land cover variables to jaguars’ space use^33^. We considered as significant relationships those with *p* values < 0.1 assuming that a marginal significant effect is reasonable for our study design following Amrhein et al.^47^. The models were carried through the package piecewiseSEM (v.2.0.2^41^) implemented for R statistical software^40^.

## Results

We observed that the frequency of jaguars tended to be higher as the distance to paved roads increased until a value of 5 km, after which it started to decrease (Fig. 2). The relationship between the frequency of jaguars and distance to unpaved roads was not very clear. We then assumed that 5 km correspond to a road-effect zone for jaguars^48,49^ both for paved and unpaved roads and the analyses were performed only for the cells located within 5 km of the roads.

The value of frequency of jaguars varied little between models (for most models values were between 0.002 and 0.21, see Supplementary Fig. S3 online for information on the distribution of each variable). Path analyses for the global, males’ and females’ models revealed that neither paved nor unpaved roads had significant direct effects on jaguars’ space use (Fig. 3, Supplementary Tables S3 and S4 online). However, both paved and unpaved roads had indirect effects on jaguars´ space use through their negative association with forest and open areas and their positive association with urban areas. The indirect effects of paved roads via forest on jaguars in the global model (Fig. 3a) was also observed for both males (Fig. 3c) and females (Fig. 3e), while the indirect negative effect of paved roads via urban areas at the global model (Fig. 3a) was replicated only for females (Fig. 3e). The indirect negative effects of unpaved roads via open areas (Fig. 3b) was also observed on males (Fig. 3d), but not on females (Fig. 3f); the indirect effect of unpaved roads via urban areas (Fig. 3b) was also found on females (Fig. 3f). The indirect effect of unpaved roads on males via urban areas was positive, i.e., the frequency of male jaguars was higher in cells near urban areas associated with unpaved roads (Fig. 3d).

Land cover had significant direct effects on jaguars in the global model (Fig. 3a, b) as well as on males (Fig. 3c, d) and females (Fig. 3e, f). As expected, forests and open areas favoured jaguars´ space use (except on Females unpaved model, where open areas had no effect, Fig. 3f). Urban areas in turn affected space use by jaguars. Unexpectedly, farming had a positive effect on jaguars´ space use for all models and urban areas had either no effect (Fig. 3c) or a positive effect (Fig. 3d) on the frequency of males (Fig. 3b). All the direct effects of land cover variables on jaguars´ space use were higher than the indirect effects of roads (Fig. 3).

## Discussion

Our findings show that the negative effects of roads on jaguars’ space use occur indirectly, through the effects of roads on land cover. We observed that paved roads are associated with a low proportion of forest, which in turn negatively affects jaguars. Similarly, unpaved roads were associated with low proportion of open areas, which reduce jaguars´ use of space. The indirect effects of roads were also observed through the association with human-dominated areas.

The indirect effect of roads on jaguars’ space use via forest and open areas shows that the commonly reported high dependence of jaguars on natural areas^50,51^ is negatively influenced by the presence of roads, which despite being intuitive, has not been discussed in the literature. Because of jaguars’ large spatial needs, reduction and fragmentation of available habitat by roads can modify the species’ spatial patterns of movement^52^.

Avoidance of anthropic areas by jaguars has already been described^32,53^ and we have shown that this can be partly related to roads. Roads facilitate access to remote areas^5^ which favors the establishment of human settlements^9^. In turn, the growing demand of urban areas increases the need for new transport infrastructure, triggering an endless self-reinforcing cycle of human interference^54,55^. Not surprisingly, males seem to be unaffected by urban areas, which is in line with an earlier study that showed that male jaguars tend to be more adventurous than females as they moved close to areas with high human population densities^30^. The tolerance of males to anthropic areas is usually attributed to the large sizes of males’ home ranges that include ranges of many females, and to large distances travelled per day^56,57^. This adds to the fact that increasing urbanization is leaving few options for jaguars so they are forced to adapt. However, conversion of habitat tends to increase the spatial requirements of apex predators, rising conflict with humans^52,58^. A recent study that tracked a male jaguar in the vicinity of a city in Mexico reported that the core areas of the jaguar’s home range included a landfill where the jaguar opportunistically predated on dogs, raccoons, and other animals that visited the area^59^. More recently, a male jaguar became famous in Brazil after traveling through different places within a city, including a church, a hotel’s parking lot, industrial neighborhood streets, and the backyard of a residence to feed on chickens, and intervention by environmental agencies was necessary to relocate the individual^60^.

The effects of roads on space use by felids have been reported in various species, including jaguars^30,61,62^. For example, jaguars’ home ranges have been found to increase with the increase of road density^63^. However, the response to roads by felids appears to be scale-dependent. For instance, cougars (*Puma concolor*) and bobcats (*Lynx rufus*) in southern California selected against roaded areas in home range selection, but they did not avoid roads in movements within home ranges^64,65^. Since we analyzed the areas immediately surrounding jaguar’s occurrences, it is not possible to make inferences about home range selection, thus, our inferences are limited to jaguars´ response to roads and land cover within their territories, corresponding to the third-order selection of resources^66^. At this scale, our results for jaguars are similar to those for cougars and bobcats^64,65^. Morato et al.^38^ studied jaguars in most of the sites we analyzed here, and also found that roads had no effect on resource selection of jaguars at the scales of home range and foraging, i.e., third and fourth-order resource selection, respectively^66^. This is not surprising since road mortality of jaguars is commonly reported^67,68^ and some carnivores can use roads as travel corridors^18,69^. In contrast, Colchero et al.^30^ modeled the movement of jaguars and found that the jaguars avoided moving close to roads within their home ranges in the Mayan Forests of Mexico and Guatemala. None of these studies, however, discussed whether the behavior of the species studied was related to the road disturbance or due to the habitat in the surroundings. We took the analysis a step further and showed that the effects of roads on jaguars can be mainly indirect, and operate via the interaction of sex and habitat type. These findings clarify the results from previous work and add to the literature about space use by jaguars in relation to roads (e.g.^38,63^).

We have disentangled direct and indirect effects of roads on jaguars, which can be a powerful tool to appropriately prioritize preventive and adaptive management actions for conservation^70^, but there are some limitations that need to be considered. First, we assumed that roads are the main drivers of land cover changes, which is theoretically sound^9^, but other landscape features may play a role as well, such as mines, dams and other human constructions^71^. Likewise, other factors may also influence jaguars´ space use, such as prey availability^27^ and movement of conspecifics^72^. Second, information about traffic volume could also help clarify the direct effects of roads^12^; the lack of detailed and systematic traffic data is one of the main limitations in many road ecology studies. Finally, the positive association of farming to jaguars´ space use may be related to the nature of our data layer; farming included both agriculture and pasture areas where livestock occur and it has been reported that livestock may attract jaguars^73^, but see^72^. More specific analysis will be necessary to better understand these relationships.

The growing plans to expand the road network in Brazil^74^ urgently require an evaluation of all the potential environmental impacts to properly balance development and conservation^75^. The results presented here are useful to guide prevention and mitigation actions for jaguars. Our findings indicate a lack of road avoidance behavior at the level of home range, which makes road mortality an important concern for jaguar conservation considering existing and planned future roads^76^. Since additional mortality may become a critical threat to a species with low reproduction rates, in particular when combined with other sources of non-natural mortality^77,78^, it is an important recommendation to identify areas of high road-kill rates and areas of movement corridors crossed by roads to implement effective measures to avoid road mortality and provide safe crossings^79–81^. Also, our study highlighted that substantial efforts should be made to control and prevent deforestation^82^ and urban sprawl^55^ due to roads, for example, by funding studies that simulate the impacts of planned roads on the landscapes still inhabited by jaguars^83^. Unfortunately, jaguar populations most at risk to disappear in Brazil are those in areas that have the highest road densities^84,85^, which have promoted deforestation and urban expansion^86^ and where road mortality has been reported as an imminent threat^68^. Given the high vulnerability of many jaguar populations in Brazil and other frequent threats they face throughout their range^58^, efforts by scientists, road managers, and government environmental agencies need to be increased and joined to be able to minimize the negative effects of roads before they exceed jaguars´ ability to maintain their populations and ecosystemic relationships.

## Supplementary Information

Supplementary Information 1.

Supplementary Table S1.
