# Supplementary material for: Direct and indirect effects of roads on space use by jaguars in Brazil
Source: Sci Rep. 2021 Nov 19;11:22617. doi: 10.1038/s41598-021-01936-6 (PMC8604938; doi:10.1038/s41598-021-01936-6)
Supplement: Supplementary file 1 — Supplementary Information 1. [file 41598_2021_1936_MOESM1_ESM.pdf]

Scientific Reports

SUPLLEMENTARY MATERIAL

Direct and indirect effects of roads on space use by jaguars in Brazil

Rafaela Cobucci Cerqueira[1,*], Oscar Rodríguez de Rivera[2], Jochen A. G. Jaeger[3], Clara Grilo[1,4]

[1] Departamento de Biologia, Universidade Federal de Lavras, Câmpus Universitário, Caixa Postal 3037, CEP 37200-000, Lavras, Minas Gerais, Brazil

[2] School of Mathematics, Statistics and Actuarial Science, University of Kent, Sibson, Park Wood Rd, Canterbury CT2 7FS, U.K.

[3] Department of Geography, Planning and Environment, Concordia University Montreal, 1455 de Maisonneuve Blvd. W., Suite H1255, Montréal, QC H3G 1M8, Canada

[4] CESAM - Centro de Estudos do Ambiente e do Mar, Departamento de Biologia Animal, Faculdade de Ciências, Universidade de Lisboa, 1749-016 Lisboa, Portugal.

[*] Corresponding author: rafaelacobucicerqueira@gmail.com

Supplementary figures

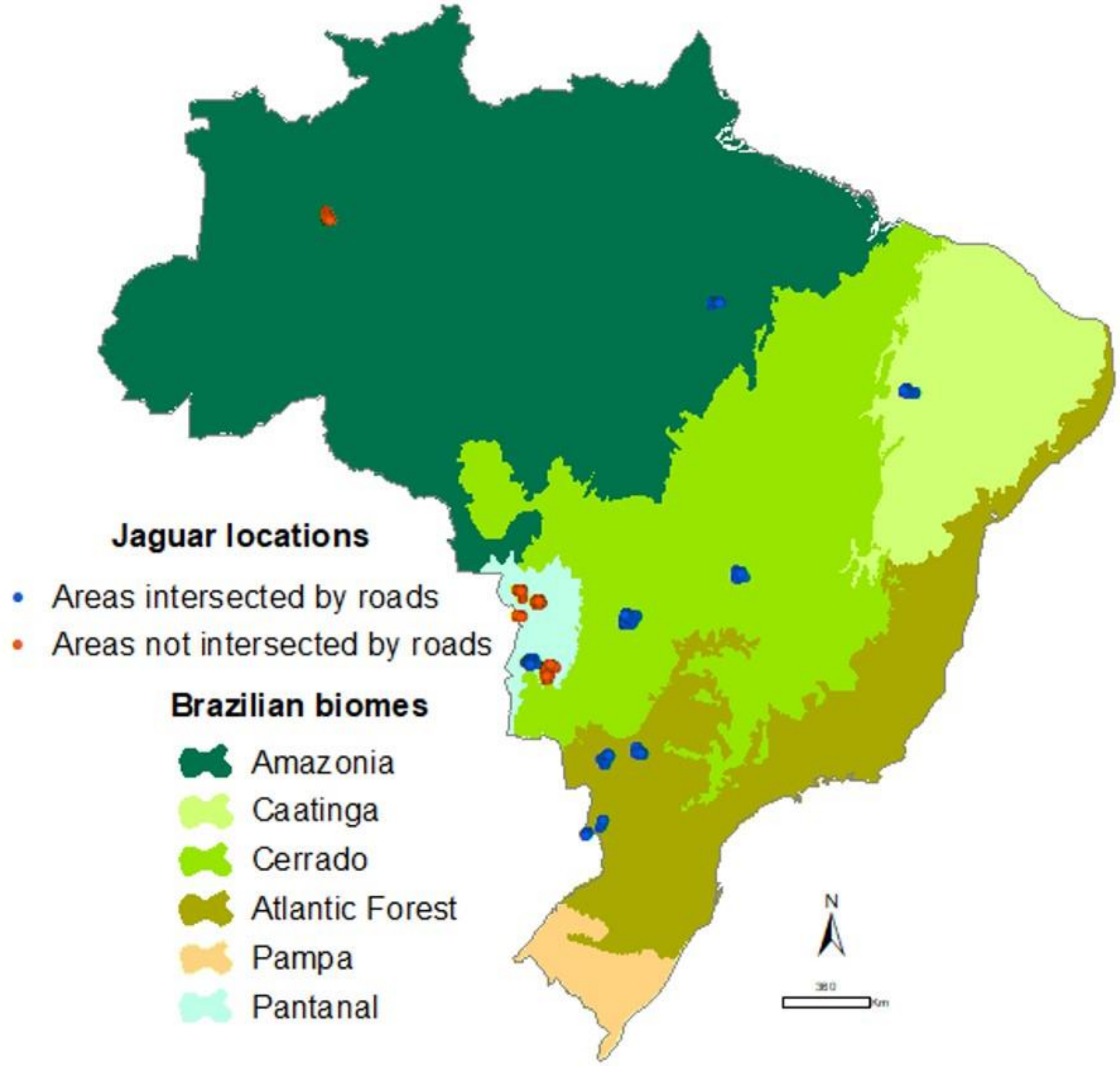

Figure S1: Jaguars locations distributed in 15 areas in different biomes in Brazil. Two areas are located in the Amazon, two in the Cerrado Biome, one in the Caatinga, five in the Pantanal, one in the transition of Cerrado-Pantanal, and four in the Atlanic Forest.

Global paved

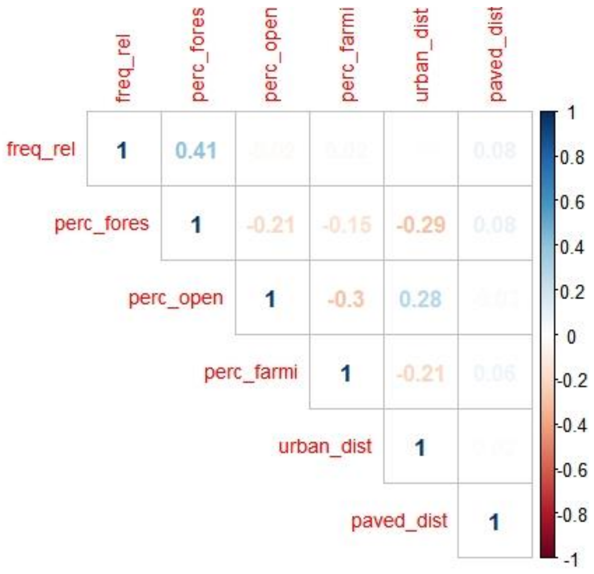

Global unpaved

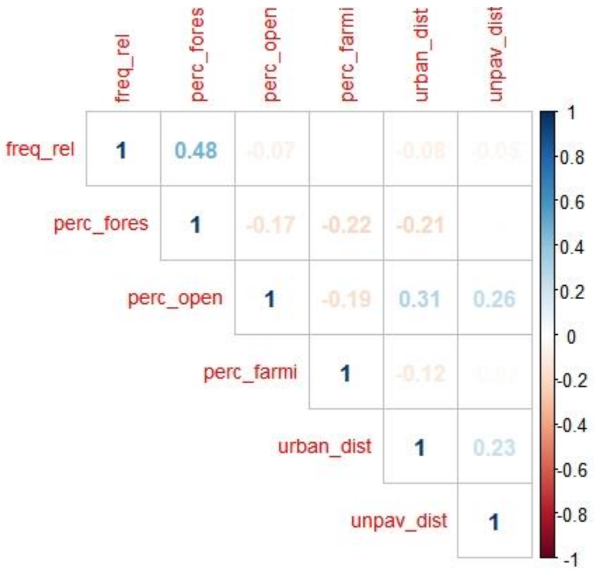

Males paved

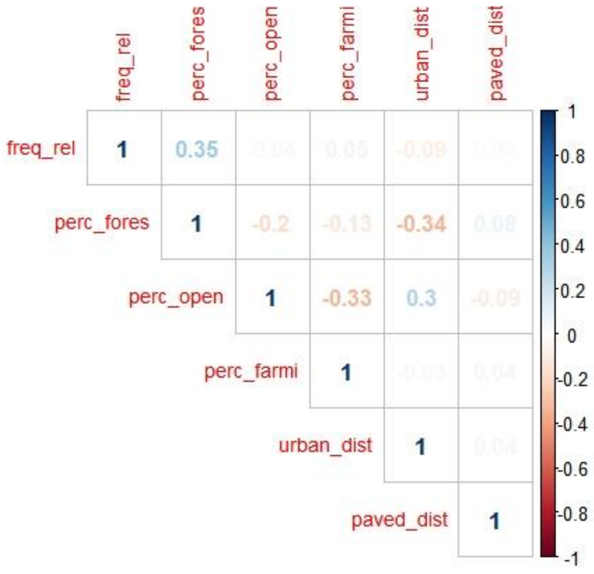

Males unpaved

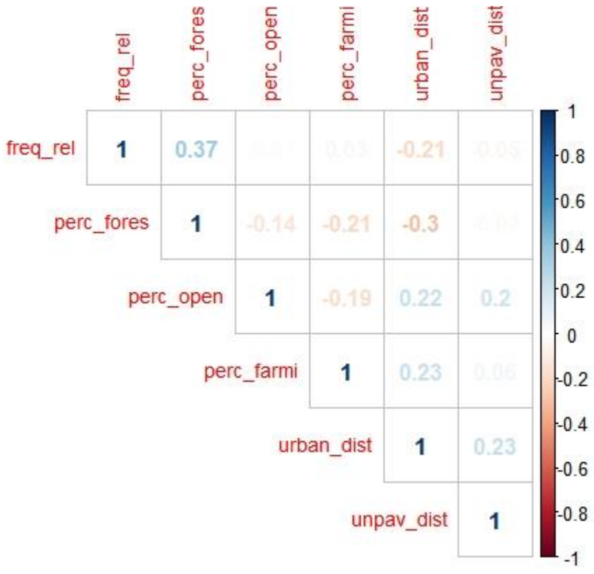

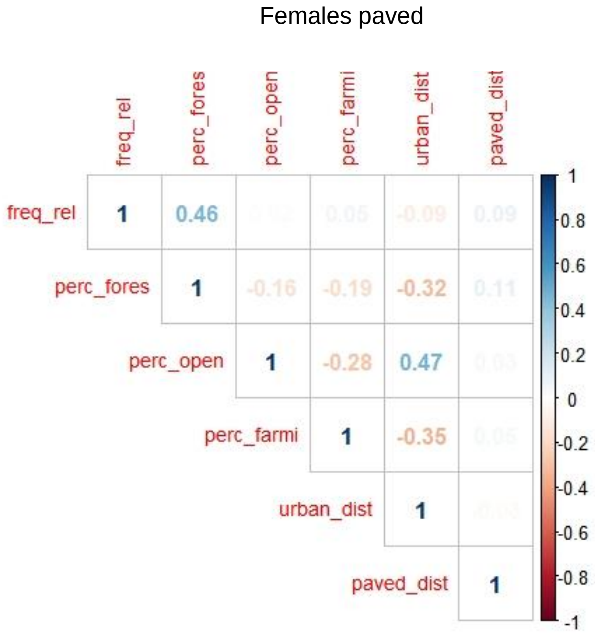

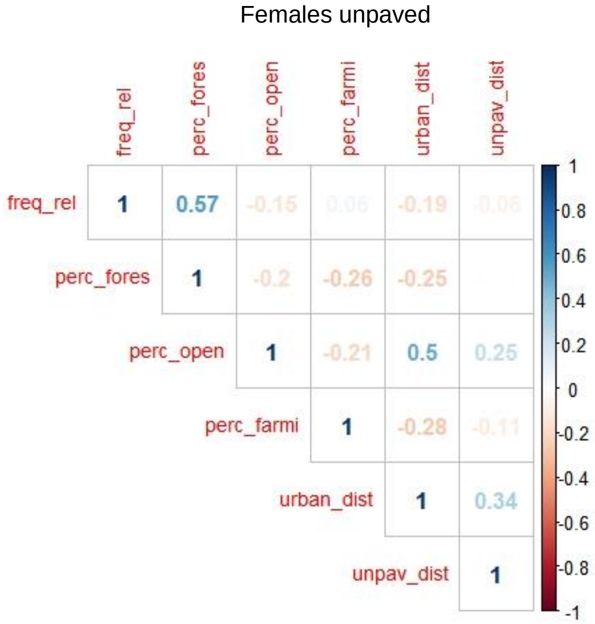

Figure S2: Results of Spearman correlation test. The results are presented for each model separately because they are different from each other spatially: Male and female locations are different and all the other variables were calculated in grid cells based on jaguar locations. freq_rel: jaguar frequency, perc_fores: % of forest, perc_opec: % of open areas, perc_farmi: % of farming, urban_dist: distance to the nearest urban area, pav/unpav_dist: distance to the nearest paved/unpaved road.

Global model: paved roads

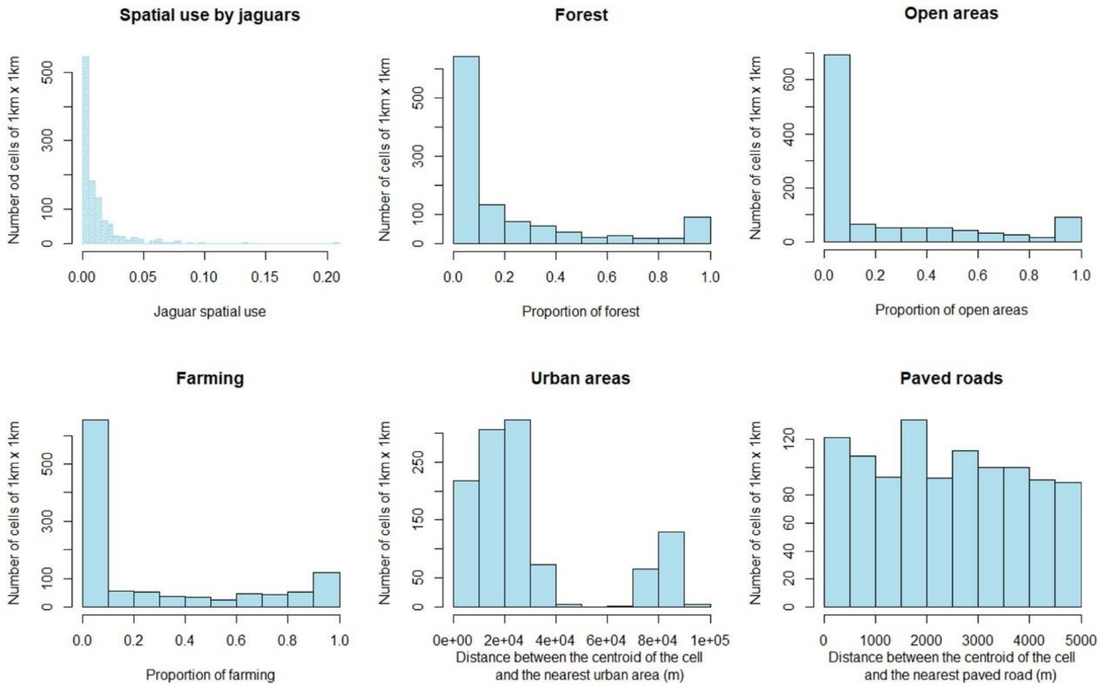

Males: paved roads

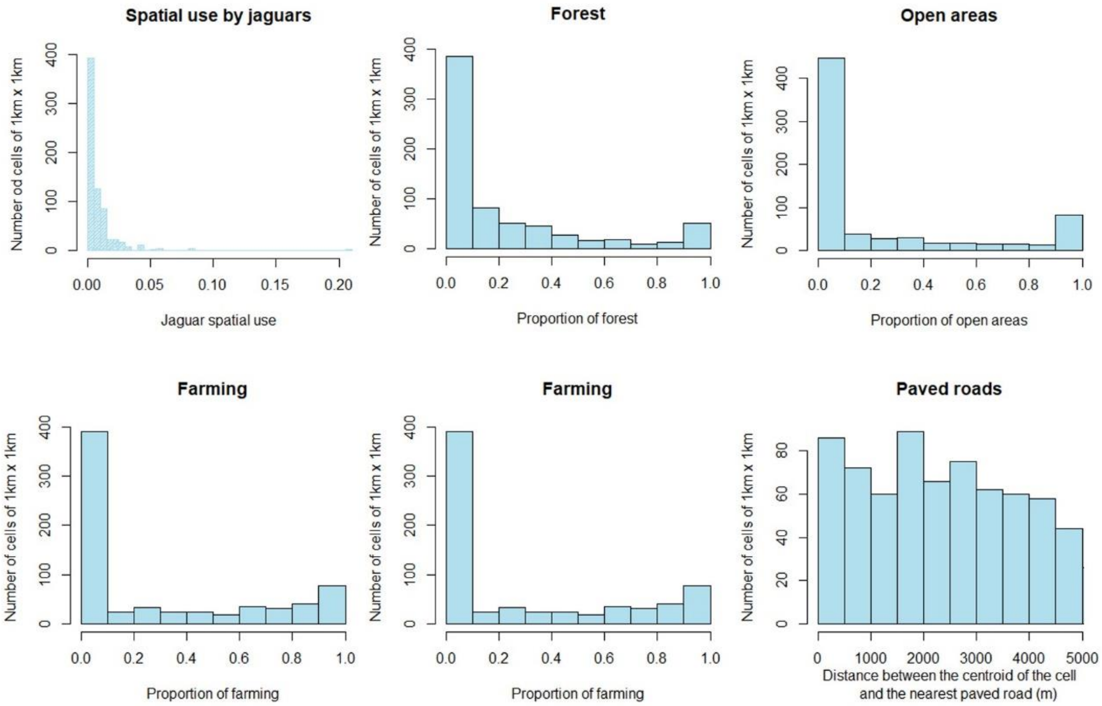

Females: paved roads

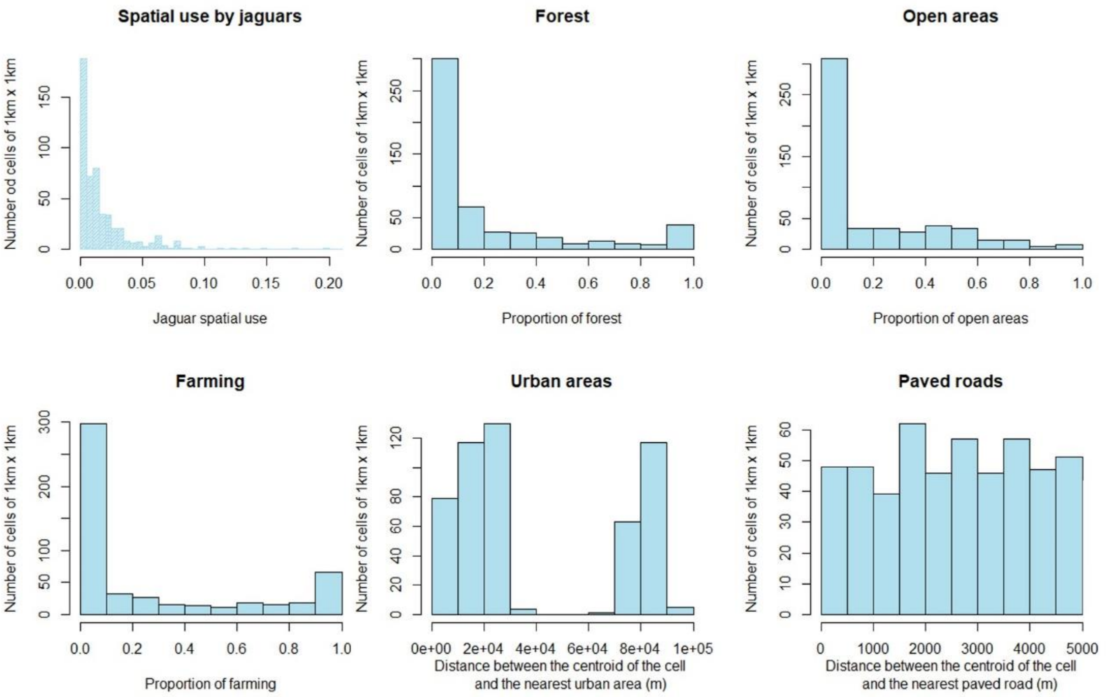

Global model: unpaved roads

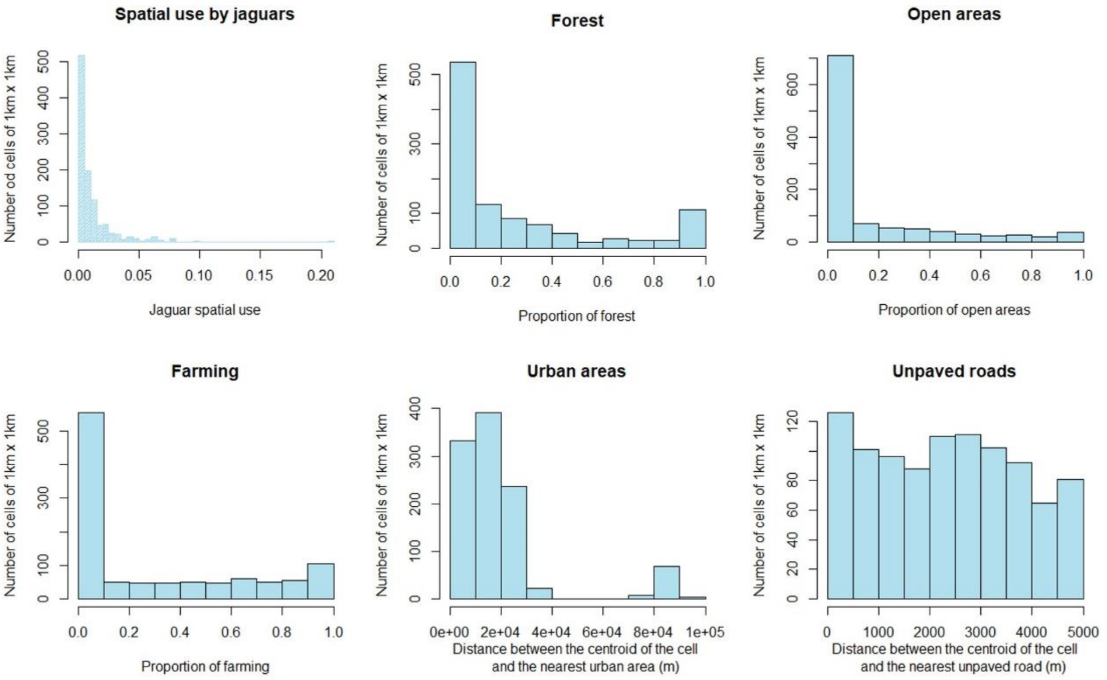

Males: unpaved roads

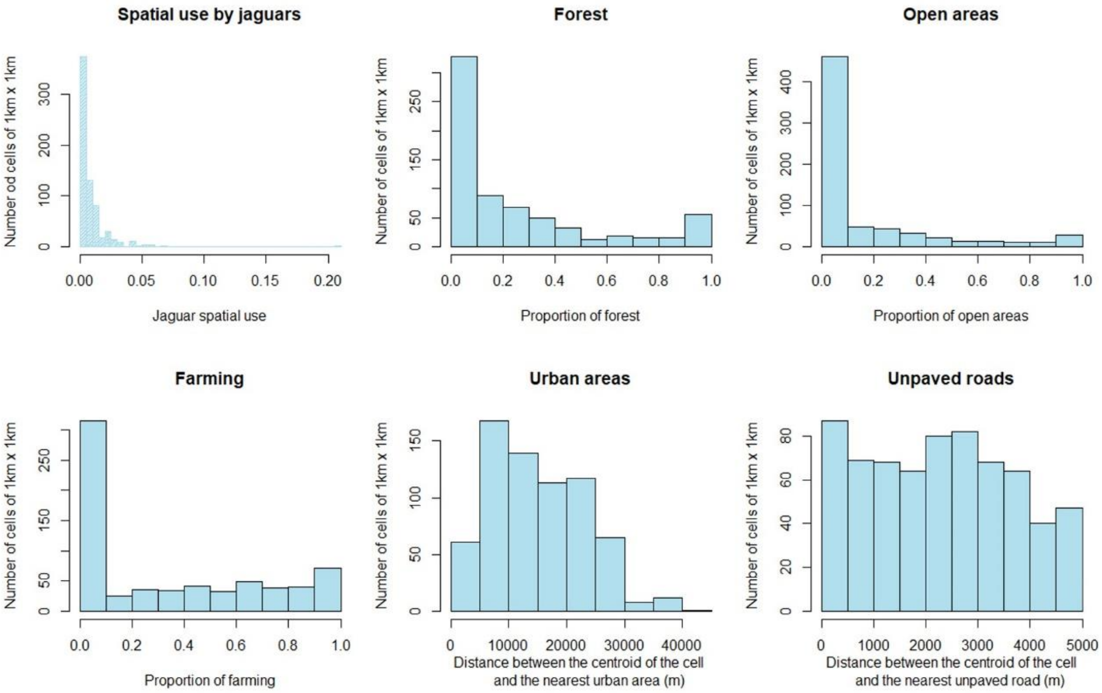

Females: unpaved roads

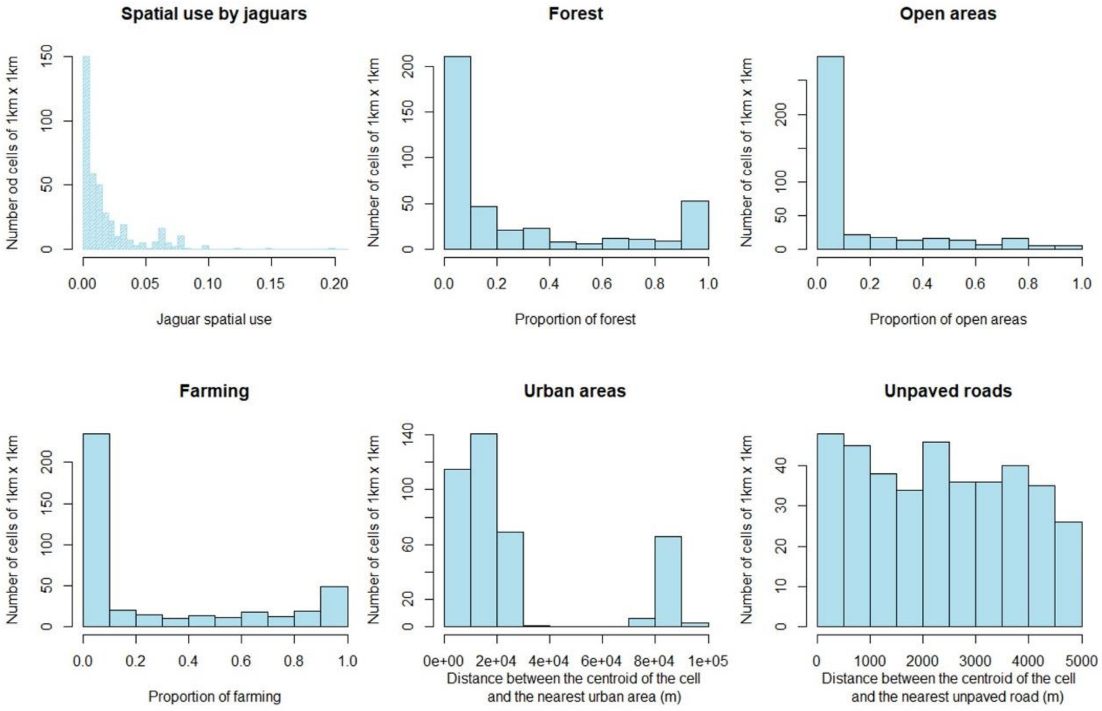

Figure S3: Histograms for the models of paved and unpaved roads for the global model, for males and females: Jaguars’ space use (number of jaguar locations per day); percentages of forest, open areas, and farming; distance to the nearest urban area, paved road and unpaved road.

Supplementary tables

Table S1: Individual jaguar locations from the 15 areas, name of the project in which the animals were recorded (Morato et al. 2018b), sex, year of recording, ID of the area to which the location belongs and description of the area (if it is intersected by paved and unpaved roads or not). *(File attached separately as TableS1.xlsx)*

Table S2: Hypothesized associations between roads (distance), land cover variables, and jaguar frequency, and possible mechanisms explaining these associations.

**Causal variable**	**Affected variable**	**Expected relationship***	**Expected effect***	**Hypothesized mechanism**	**Equation**
Distance to roads (paved or unpaved)	Forest	Positive	Negative	The larger the distance to roads, the higher the proportion of natural areas (forest and open areas). Roads are key in deforestation processes because they facilitate reductions in the amount of natural areas by vegetation clearance due to road construction and use (Fearnside et al. 2006).	forest ~ paved roads<br>forest ~ unpaved roads
Distance to roads (paved or unpaved)	Open areas	Positive	Negative		open ~ paved roads<br>open ~ unpaved roads
Distance to roads (paved or unpaved)	Farming	Negative	Positive	The shorter the distance to roads, the higher the proportion of farming. Roads facilitate rural livelihoods (Laurance et al. 2014).	farming ~ paved roads<br>farming ~ unpaved roads

Distance to roads (paved or unpaved)	Distance to urban areas	Positive	Positive	The shorter the distance to roads, the shorter the distance to urban areas. The presence of roads facilitates urbanization (Laurance et al. 2009).	urban ~ paved roads<br>urban ~ unpaved roads
Distance to roads (paved or unpaved)	Jaguar spatial use	Positive	Negative	The larger the distance to roads, the higher the spatial use by jaguars. Road avoidance behavior has been reported for jaguars by some studies, suggesting that they tend to occur far from roads (Conde et al. 2010, Colchero et al. 2010).	frequency of jaguars ~ paved roads + forest + open + farming + urban
Forest	Jaguar spatial use	Positive	Positive	The higher the proportion of natural areas (forest and open areas), the higher the spatial use by jaguars. Jaguars are highly dependent on native forest (De Angelo et al. 2011), and forest and open areas are considered important habitats (Zeilhofer et al. 2014). Their occurrence is	frequency of jaguars ~unpaved roads + forest + open + farming + urban
Open areas	Jaguar spatial use	Positive	Positive		

				negatively affected by habitat loss (Hatten et al. 2005; Morrison et al. 2007).
Farming	Jaguar spatial use	Negative	Negative	The higher the proportion of farming, the lower the spatial use by jaguars. Human land use and presence, such as in agriculture land, pastures, and farms, have negative effects on jaguar presence (De Angelo et al. 2011).
Distance to urban areas	Jaguar spatial use	Positive	Negative	The larger the distance to urban areas, the higher spatial use by jaguars. Jaguars´ presence is negatively affected by human presence and human population density (De Angelo et al. 2011).

* ‘Expected relationship’ is related to the relationship between the variables taking into account the units of measurement of the variables, and ‘expected effect’ is related to the effect of the causal variables on the affected variable. For example, the expected relationship between (distance to) roads and proportion of forest is positive because the unit of measurement of road is distance and the unit of measurement of forest is percentage (see Section 2.2 of Methods), and we expect that the higher the distance to roads, the higher the proportion forest, which corresponds to a negative effect of roads on forest.

Table S3: Statistical results of path analyses for the models for paved roads for global model, for males, and females. Standardized effects of explanatory variables on forest, open areas, farming, urban areas, and frequency of jaguars. (○ *p*–value < 0.1, * *p*–value < 0.05, and ***p*–value < 0.01).

Response	Explanatory	Global		Males		Females	
		Estimate ± SD	*p*-value	Estimate ± SD	*p*-value	Estimate ± SD	*p*-value
Forest	Distance to paved roads	0.0412 ± 0.0204	0.0435*	0.0525 ± 0.027	0.0514○	0.0572 ± 0.0329	0.0815○
Open areas	Distanace to paved roads	0.0155 ± 0.0167	0.3525	0.0007 ± 0.0214	0.9744	0.0185 ± 0.0235	0.431
Farming	Distance to paved roads	0.0169 ± 0.0189	0.3724	0.0039 ± 0.0256	0.8793	0.027 ± 0.0269	0.3164
Urban areas	Distance to paved roads	0.017 ± 0.0042	0.0001**	0.024 ± 0.0109	0.0282*	0.0249 ± 0.0066	0.0002**

Frequency of jaguars	Distance to paved roads	0.0103 ± 0.0248	0.6785	0.0048 ± 0.0315	0.879	-0.0003 ± 0.0345	0.9939
	Forest	0.3701 ± 0.0288	0**	0.273 ± 0.0353	0**	0.419 ± 0.0408	0**
	Open areas	0.0979 ± 0.0271	0.0003**	0.0905 ± 0.0353	0.0104*	0.1116 ± 0.0393	0.0045**
	Farming	0.145 ± 0.0272	0**	0.1138 ± 0.0338	0.0008**	0.222 ± 0.0396	0**
	Distance to urban areas	0.1138 ± 0.0273	0**	0.0337 ± 0.0344	0.3275	0.1145 ± 0.0431	0.0079**

Table S4: Statistical results of path analyses for the models for unpaved roads for the global model, for males, and females. Standardized effects of explanatory variables on forest, open areas, farming, urban areas, and frequency of jaguars. (○ *p*–value < 0.1, * *p*–value < 0.05, and ***p*–value < 0.01).

Response	Explanatory	Global		Males		Females	
		**Estimate ± SD**	***p*-value**	**Estimate ± SD**	***p*-value**	**Estimate ± SD**	***p*-value**
Forest	Distance to unpaved roads	0.0189 ± 0.0213	0.3759	0.0197 ± 0.0275	0.4745	0.0152 ± 0.0376	0.6867
Open areas	Distance to unpaved roads	0.0896 ± 0.0202	0**	0.0859 ± 0.0272	0.0016**	0.0589 ± 0.0279	0.0344*
Farming	Distance to unpaved roads	-0.0082 ± 0.0219	0.7097	0.0217 ± 0.0279	0.4363	-0.0006 ± 0.0342	0.9864
Urban areas	Distance to unpaved roads	0.0432 ± 0.0071	0**	0.0583 ± 0.0101	0**	0.0651 ± 0.0101	0**

Frequency of jaguars	Distance to unpaved roads	-0.0417 ± 0.0254	0.1008	-0.0155 ± 0.0325	0.6339	-0.0517 ± 0.0383	0.1769
	Forest	0.3879 ± 0.028	0**	0.2648 ± 0.0344	0**	0.5146 ± 0.0436	0**
	Open areas	0.0758 ± 0.0266	0.0044**	0.1032 ± 0.0336	0.0021**	0.0494 ± 0.0419	0.2378
	Farming	0.1471 ± 0.0256	0**	0.1324 ± 0.0335	0.0001**	0.2581 ± 0.0404	0**
	Distance to urban areas	0.0479 ± 0.0263	0.0684○	-0.0633 ± 0.0353	0.0727○	0.0944 ± 0.0454	0.0375*

Supplementary text

Text S1: Details on simultaneous autoregressive (SAR) models and R-squares used in the piecewise SEM analysis

SAR models augment the standard linear regression model with an additional term that incorporates the spatial autocorrelation structure of a given data set. This additional term is implemented with a 'spatial weights matrix' where the neighbourhood of each location and the weight of each neighbour need to be defined (Anselin & Bera, 1998; Fortin & Dale, 2005). We have applied the SAR lagged model that assumes that the autoregressive process occurs only in the response variable ('inherent spatial autocorrelation'), and thus includes a term (ρW) for the spatial autocorrelation in the response variable Y, but also the standard term for the explanatory variables and errors ($X\beta + e$) as used in an ordinary least squares regression. The SAR lag takes the following expression:

$$Y = \rho WY \ + X\beta + e$$

Where ρ is the autoregression coefficient, W is the spatial weights matrix, β is a vector representing the slopes associated with the explanatory variables in the original predictor matrix X, and e represents the (spatially) independent errors (Kissling & Carl, 2008).

Many methods have been used to evaluate model fit in non-linear regressions, including 'pseudo-R^2' measures of explained variance (Nagelkerke, 1991; Cox & Snell, 1989). In our case the model is fitted by maximum likelihood, so likelihood-based measures are more appropriate. Also, the model is non-linear in the spatial coefficient. For that reason we have used Generalised R-squared, also known as the Nagelkerke or Craig and Uhler R^2

(Nagelkerke, 1991), which is an extension of the R^2 measure that can be applied to general regression models. It compares the likelihood of the fitted model (L_M) to the likelihood of the intercept-only (constant) model (L_0) and it is scaled to have a maximum of 1:

$$GeneralisedR\text{-}squared = 1 - \left(\frac{L_0}{L_M}\right)^{\left(\frac{2}{n}\right)}$$

A value of 1 indicates a perfect model; a value of 0 indicates that a model is no better than a constant. The measure simplifies to the traditional R-square for continuous normal responses in the standard least squares setting.

REFERENCES

Anselin L, Bera AK (1998) Spatial dependence in linear regression models with an introduction to spatial econometrics. In: Ullah A, Giles DEA (eds) Handbook of Applied Economic Statistics. Marcel Dekker, New York, pp. 237–289.

Colchero F, Conde DA, Manterola, Chávez C, Rivera A, Ceballos G (2011) Jaguars on the move: modeling movement to mitigate fragmentation from road expansion in the Mayan Forest. Anim Conserv 4: 158–166. https://doi.org/10.1111/j.1469-1795.2010.00406.x

Conde DA, Colchero F, Zarza H, Christensen NL, Sexton JO, Manterola C, Chávez C, Rivera A, Azuara D, Ceballos G (2010) Sex matters: modeling male and female habitat differences for jaguar conservation. Biol Conserv 143 (9): 1980-1988. https://doi.org/10.1016/j.biocon.2010.04.049

Cox DR, Snell EJ (1989). The Analysis of Binary Data. Chapman and Hall, London.

De Angelo C, Paviolo A, Di Bitetti M (2011) Differential impact of landscape transformation on pumas (*Puma concolor*) and jaguars (*Panthera onca*) in the Upper Paraná Atlantic Forest. Divers Distrib 17: 422-436. http://doi.org/10.1111/j.1472-4642.2011.00746.x

Fearnside PM, de Alencastro Graça PML (2006) BR-319: Brazil's Manaus-Porto Velho Highway and the Potential Impact of Linking the Arc of Deforestation to Central Amazonia. Environ Manage 38: 705–716. https://doi.org/10.1007/s00267-005-0295-y

Fortin MJ, Dale MRT (2005) Spatial analysis - a guide for ecologists. Cambridge University Press, Cambridge.

Hatten JR, Averill-Murray A, Van Pelt WE (2005) A spatial model of potential jaguar habitat in Arizona. J of Wildl Manag 69 (3): 1024-1033. https://doi.org/10.2193/0022-541X(2005)069[1024:ASMOPJ]2.0.CO;2

Kissling WD, Carl G (2008) Spatial autocorrelation and the selection of simultaneous autoregressive models. Glob Ecol Biogeogr 17(1): 59-71. https://doi.org/10.1111/j.1466-8238.2007.00334.x

Laurance WF, Goosem M, Laurance SGW (2009) Impacts of roads and linear clearings on tropical forests. Trends Ecol Evol 24: 659 - 669. https://doi.org/10.1016/j.tree.2009.06.009

Laurance WF, Clements GR, Sloan S, O'Connell CS, Mueller ND, Goosem M, Venter O, Edwards DP, Phalan B, Balmford A, Van Der Ree R, Arrea IB (2014). A global strategy for road building. Nature 513:229 - 232. https://doi.org/10.1038/nature13717

Morato RG, Thompson JJ, Paviolo A, de La Torre JA, Lima F, McBride RT, Paula RC, Cullen L, Silveira L et al. (2018b) Jaguar movement database: A GPS-based movement dataset of an apex predator in the Neotropics. Ecology 99: 1691-1691. http://doi.org/10.1002/ecy.2379

Morrison JC, Sechrest W, Dinerstein E, Wilcove DS, Lamoreux JF (2007) Persistence of large mammal faunas as indicators of global human impacts. J Mammal, 88(6): 1363 - 1380. https://doi.org/10.1644/06-MAMM-A-124R2.1

Nagelkerke NJ (1991) A note on a general definition of the coefficient of determination. Biometrika 78(3): 691 - 692. http://doi.org/10.1093/biomet/78.3.691

Zeilhofer P, Cezar A, Tôrres NM, Jácomo ATA, Silveira L (2014) Jaguar Panthera onca Habitat Modeling in Landscapes Facing High Land-use Transformation Pressure - Findings from Mato Grosso, Brazil. Biotropica 46(1): 98-105. https://doi.org/10.1111/btp.12074
